# Changes in the nitric oxide pathway of the pulmonary vasculature after exposure to hypoxia in swine model of neonatal pulmonary vascular disease

**DOI:** 10.14814/phy2.13889

**Published:** 2018-10-29

**Authors:** Daphne P. M. de Wijs‐Meijler, Dirk J. Duncker, A. H. Jan Danser, Irwin K. M. Reiss, Daphne Merkus

**Affiliations:** ^1^ Division of Experimental Cardiology Department of Cardiology University Medical Center Rotterdam Erasmus MC Rotterdam The Netherlands; ^2^ Division of Neonatology Department of Pediatrics Sophia Children's Hospital Erasmus MC University Medical Center Rotterdam Rotterdam The Netherlands; ^3^ Division of Pharmacology Department of Internal Medicine Erasmus MC University Medical Center Rotterdam Rotterdam The Netherlands

**Keywords:** Exercise, hypoxia, nitric oxide, pulmonary vascular disease, soluble guanylyl cyclase

## Abstract

Neonatal pulmonary vascular disease (PVD) is increasingly recognized as a disease that complicates the cardiopulmonary adaptations after birth and predisposes to long‐term cardiopulmonary disease. There is growing evidence that PVD is associated with disruptions in the nitric oxide (NO)‐cGMP‐phosphodiesterase 5 (PDE5) pathway. Examination of the functionality of different parts of this pathway is required for better understanding of the pathogenesis of neonatal PVD. For this purpose, the role of the NO‐cGMP‐PDE5 pathway in regulation of pulmonary vascular function was investigated in vivo, both at rest and during exercise, and in isolated pulmonary small arteries in vitro, in a neonatal swine model with hypoxia‐induced PVD. Endothelium‐dependent vasodilatation was impaired in piglets with hypoxia‐induced PVD both in vivo at rest and in vitro. Moreover, the responsiveness to the NO‐donor SNP was reduced in hypoxia‐exposed piglets in vivo, while the relaxation to SNP and 8‐bromo‐cyclicGMP in vitro were unaltered. Finally, PDE5 inhibition‐induced pulmonary vasodilatation was impaired in hypoxia‐exposed piglets both in vitro and in vivo at rest. During exercise, however, the pulmonary vasodilator effect of PDE5 inhibition was significantly larger in hypoxia‐exposed as compared to normoxia‐exposed piglets. In conclusion, the impaired endothelium‐dependent vasodilatation in piglets with hypoxia‐induced PVD was accompanied by reduced responsiveness to NO, potentially caused by altered sensitivity and/or activity of soluble guanylyl cyclase (sGC), resulting in an impaired cGMP production. Our findings in a newborn animal model for neonatal PVD suggests that sGC stimulators/activators may be a novel treatment strategy to alleviate neonatal PVD.

## Introduction

Dysmorphic pulmonary vascular growth and impaired alveolarization are hallmarks of the disruption of normal lung development that characterizes bronchopulmonary dysplasia (BPD) (Jobe 1999; Coalson 2003; Mourani and Abman 2013). Pulmonary vascular disease (PVD) encompasses decreased angiogenesis, abnormal vascular function with increased vasomotor tone and altered vasoreactivity, and abnormal vascular structure (vascular remodeling) with smooth muscle cell proliferation. These changes result in an increased pulmonary vascular resistance (PVR), thereby increasing the risk of pulmonary hypertension (PH) in BPD patients (Jobe 1999; Coalson 2003; Mourani and Abman 2013; Rossor and Greenough 2015). Retrospective studies have determined the incidence of BPD‐associated PH to be approximately 20–40% (Khemani et al. 2007; An et al. 2010; Slaughter et al. 2011; Weismann et al. 2017). Although most studies concerning long‐term outcome of BPD focus on respiratory function, there is increasing awareness that PVD (including PH) imposes additional morbidity, including prolonged oxygen requirements and exercise intolerance, and mortality in the already vulnerable BPD infants (Khemani et al. 2007; Slaughter et al. 2011). Therapies currently used in clinical practice for infants with PVD are supplemental oxygen therapy to avoid hypoxic pulmonary vasoconstriction (Berkelhamer et al. 2013b; Rossor and Greenough 2015), pulmonary vasodilators such as sildenafil (phosphodiesterase 5 inhibitor) and inhaled nitric oxide (iNO) (Berkelhamer et al. 2013b; Rossor and Greenough 2015), that may also limit oxidative stress caused by hyperoxia (Berkelhamer et al. 2013a; de Wijs‐Meijler et al. 2017a), However, the knowledge regarding treatment efficacy and safety of BPD‐associated PH is limited and needs to be further investigated.

Previous studies in swine showed an important role for the nitric oxide (NO)‐cGMP signaling pathway in the pathogenesis of chronic PH (Fike et al. 1998; Fike et al. 2004). Nitric oxide (NO) is produced in the vascular endothelial cells from L‐arginine by the enzyme endothelial nitric oxide synthase (eNOS), and then diffuses to the vascular smooth muscle cell. There, NO stimulates soluble guanylyl cyclase (sGC), resulting in the production of cGMP, which activates cGMP‐dependent protein kinases. Ultimately, this leads to opening of the large conductance K (BK_Ca_) channel, smooth muscle cell relaxation and thus vasodilatation (McDonald and Murad 1995). There is growing evidence that neonatal pulmonary hypertension is associated with multiple disruptions in this signaling cascade. A decreased eNOS activity and reduced vasodilator response to NO were found in different animal models for neonatal PH (North et al. 1995; Shaul et al. 1997; Tulloh et al. 1997; Fike et al. 1998, 2004; Berkenbosch et al. 2000). There are, however, only a few studies regarding the role of more downstream disruptions in the NO‐cGMP signaling pathway, including sGC ‐and cGMP‐dependent mechanisms (Steinhorn et al. 1995; Tulloh et al. 1997; Berkenbosch et al. 2000). Furthermore, no studies were performed to assess the long‐term consequences of disruptions in the NO‐cGMP signaling pathway. Therefore, examination of the functionality of different parts of this pathway is required for better understanding of the pathogenesis of BPD‐associated PH, not only in the neonatal period but also later in life.

We previously developed a neonatal swine model with hypoxia‐induced PH showing the clinical features resembling those found in patient with neonatal PVD and/or PH, in terms of pulmonary hemodynamics, abnormalities in the structure of the right ventricle and disruptions in normal lung development (vascular remodeling) (de Wijs‐Meijler et al. 2017c). This model allows 3‐weeks of follow‐up after re‐exposure to normoxia with hemodynamic measurement at rest and during exercise in awake piglets. By placing the cardiopulmonary system under stress with exercise testing, subtle dynamic abnormalities that are not apparent on conventional static tests may be revealed. Additionally, exercise testing helps to assess the severity of the disease. Consequently, the main purpose of this study was to determine the functionality of different parts of the NO‐cGMP signaling pathway in the long‐term, in vivo (at rest and during incremental exercise) and in vitro, to achieve greater understanding of the pathogenesis of neonatal PH associated with BPD (or other chronic cardiopulmonary disorders associated with hypoxia) and the long‐term sequela of damage to the developing neonatal lung.

## Methods

### Ethical approval

Studies were performed in accordance with European Directive 2010/63/EU as well as with the “Guiding Principles in the Care and Use of Laboratory Animals” as approved by the Council of the American Physiological Society, and with approval of the Animal Care Committee of the Erasmus MC Rotterdam (Protocolnumber 109‐12‐11, EMC 2702).

### In vivo animal experiments

Thirty‐four crossbred Landrace x Yorkshire piglets of either sex entered the study when they were 48 h old. Shortly after arrival, all piglets received a single dose of artificial colostrum (Colo‐active) and were placed in an incubator, in which the fraction of inspired oxygen (FiO_2_) can be regulated, for 4 weeks. Piglets assigned to the control group were exposed to a normobaric normoxic environment (FiO_2_ 21%; *N* = 16), whereas piglets assigned to the intervention group were exposed to a normobaric hypoxic environment (*N* = 18). Piglets in the Hypoxia group were exposed to FiO_2_ 10% for at least 1 week, and FiO_2_ was adjusted to higher levels (max. FiO_2_ 12%) based on clinical signs of severe pulmonary hypertension (severe dyspnea, growth retardation, septal shift on echocardiography). Piglets were fed age appropriately (Lactowean Extra, Babywean, Topwean; Denkavit, Voorthuizen, The Netherlands) and received a supplementary feed for piglets, based on egg yolk (MS Pig Pusher Oral, Schippers BV, Bladel, The Netherlands) from day 1–3, to support the immune system and to increase the vitality of the newborn piglets, especially in case of insufficient colostrum. They were weighed daily.

**Figure 1:**
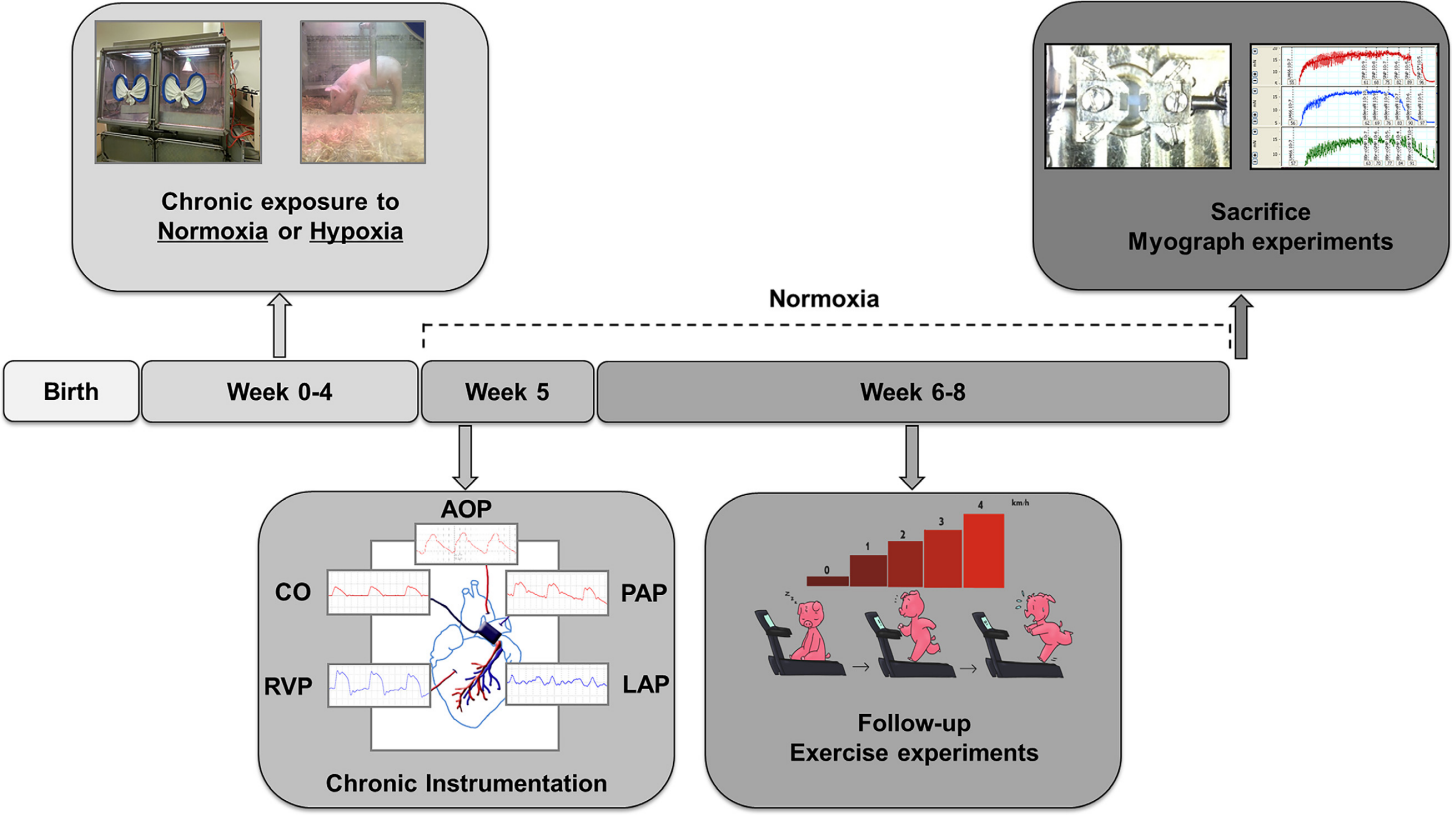
Schematic representation of the experimental design of the study.

After 4 weeks in the incubator, piglets were chronically instrumented as described below, and subsequently placed in a normoxic environment. A schematic representation of the methods is presented in Figure 1.

### Surgical procedures

Piglets were sedated with tiletamine/zolazepam (3 mg kg^−1^ i.v.), xylazine (1.75 mg kg^−1^ i.v.), and atropine (0.5 mg), intubated and ventilated with a mixture of O_2_ and N_2_ (1:2) to which 2.0% (v/v) isoflurane was added for adequate anesthesia. The depth of anesthesia was checked regularly using a pain stimulus (toe‐pinch). Due to premature death, caused by health problems related to exposure to hypoxia (*N* = 6), only 28 piglets (Normoxia, *N* = 16;, Hypoxia *N* = 12) were instrumented under sterile conditions as previously described (De Wijs‐Meijler et al. 2016). Briefly, the chest was opened via the fourth left intercostal space and fluid‐filled polyvinylchloride catheters were inserted into the aortic arch, pulmonary artery, left atrium, and right ventricle for pressure measurements. Furthermore, these catheters were used for blood sampling as well as for the infusion of drugs. In a subset of animals (*N* = 13), a flow probe (14–16 mm, Transonic Systems) was positioned around the pulmonary artery for measurement of cardiac output. Catheters and electrical wires were tunnelled subcutaneously to the back and the chest was closed in layers. All animals were placed in a normoxic environment and were allowed to recover, receiving analgesia (Buprenorphine 0.015mg kg^−1^ i.m., Fentanyl slow‐release patch 6 *μ*g h^−1^) and antibiotic prophylaxis (Augmentin 25/5 mg kg^−1^ i.v.) for 7 days. The catheters were flushed daily with heparinized saline (1000–5000 IE mL^−1^). Five piglets died during or shortly after surgery, and four piglets were not able to perform exercise experiments due to limb infection. Finally, 19 piglets (Normoxia, *N* = 10 (six female, four male); Hypoxia, *N* = 9 (five female, four male)) were included for analysis.

### Experimental protocols; agonist‐induced vasodilatation

Studies were performed 10 ± 1 days after surgery in both groups. Fluid‐filled pressure transducers were positioned on the back of the animals and calibrated at midchest level. Baseline hemodynamic measurements were obtained in resting piglets (Normoxia, *N* = 8 (six female, two male); Hypoxia, *N* = 8 (four female, four male)), consisting of heart rate, cardiac output (CO), aortic pressure (MAP), pulmonary artery pressure (PAP), left atrial pressure (LAP), and right ventricular pressure (RVP). To study the responsiveness of the vascular beds to nitric oxide (NO), we determined the hemodynamic responses to the endothelium‐independent exogenous NO‐donor sodium nitroprusside (SNP; 0.5–5 *μ*g kg^−1^ min^−1^, i.v.) in resting swine.

### Experimental protocols; exercise experiments

Control exercise trials were performed 9 ± 1 days after surgery in both groups with piglets exercising on a motor‐driven treadmill. Fluid‐filled pressure transducers were positioned on the back of the animals and calibrated at midchest level. With piglets (Normoxia, *N* = 10 (six female, four male); Hypoxia, *N* = 9 (five female, four male)) standing on the treadmill, resting hemodynamic measurements, consisting of heart rate, cardiac output (CO), MAP, PAP, LAP, and RVP were obtained. Rectal temperature was measured, and arterial and mixed venous blood samples were collected. Subsequently, a four‐stage treadmill exercise protocol was started (1–4 km h^−1^); each exercise stage lasted 3 min. Hemodynamic parameters were continuously recorded and blood samples were collected during the last 45 sec of each exercise stage, at a time when hemodynamics had reached a steady state (De Wijs‐Meijler et al. 2016). After completing the exercise protocol, animals were allowed to rest on the treadmill. After 90 min of rest, two different protocols (see below) were performed in a subset of swine, on different days and in random order. The number of swine in each protocol, as well as overlap between protocols, are shown in Table 1. Excellent reproducibility of consecutive exercise trials has been reported previously (Duncker et al. 2000).

**Table 1:** Schematic representation of the overlap of swine used in the different protocols

	Control	LNNA	EMD360527	Total
Control	**10N/9H**	9N/8H	6N/5H	
LNNA		**9N/8H**	6N/5H	
EMD360527		‐	**6N/5H**	
Total				**10N/9H**

Bold font indicates the total number of swine in each experimental protocol.

N, normoxia; H, hypoxia.

Ninety minutes after piglets had undergone a control exercise trial (as described above) the NO‐synthase inhibitor N^*ω*^‐nitro‐L‐Arginine (LNNA, Sigma) was administered at a dose of 20 mg kg^−1^ i.v. in nine normoxia‐exposed (six female, three male, 19 ± 1 days after surgery) and eight hypoxia‐exposed (five female, three male, 18 ± 1 days after surgery and re‐exposure to normoxia) piglets. Ten minutes after completion of the infusion, resting measurements were obtained and the four‐stage exercise protocol was repeated (Houweling et al. 2005).

On a different day (16 ± 2 days after surgery for normoxia animals and 14 ± 1 days after surgery and re‐exposure to normoxia for the hypoxia‐exposed animals), the exercise protocol was repeated, but during the second exercise protocol the phosphodiesterase‐5 (PDE5) inhibitor EMD360527 (a gift from Merck, Darmstadt, Germany) was infused continuously in a dose of 300 *μ*g kg^−1 ^min^−1^ i.v. in six normoxia‐exposed (two female, four male) and five hypoxia‐exposed (three female, two male) piglets. Ten minutes after starting the infusion, resting measurements were obtained and the four‐stage exercise protocol was performed (Houweling et al. 2012; Zhou et al. 2014).

### In vitro myograph experiments

At the end of the study (4–6 weeks after re‐exposure to normoxia), all piglets (Normoxia, *N* = 10; Hypoxia, *N* = 9) were reanesthetized and killed by inducing cardiac arrest using electromechanical dissociation of the heart. Right lungs were immediately excised and pulmonary small arteries (diameter ≈ 300 *μ*m) were dissected out from the lower lung lobe and stored overnight placed in cold, oxygenated Krebs bicarbonate solution of the following composition (in mmol/L): NaCl 118, KCl 4.7, CaCl_2_ 2.5, MgSO_4_ 1.2, KH_2_PO_4_ 1.2, NaHCO_3_ 25, and glucose 8.3; pH 7.4. The next day, pulmonary small arteries were cut into segments of ~2 mm length and mounted in microvascular myographs (Danish MyoTechnology) with separated 6 mL organ baths containing Krebs bicarbonate solution aerated with 95% O_2_‐5% CO_2_ and maintained at 37 ° C (Mulvany and Halpern 1977). Changes in contractile force were recorded with a Harvard isometric transducer. Following a 30‐min stabilization period, a length‐tension curve was constructed and the internal diameter of the pulmonary small arteries was set to a tension equivalent to 0.9 times the estimated diameter at 20 mmHg effective transmural pressure. Vessels were then exposed to 30 mmol/L KCl twice. Endothelial integrity of pulmonary arteries was verified by observing dilation to 10 nmol/L substance P after precontraction with 100 nmol/L of the stable thromboxane A_2_ analogue pyridoxalphophate‐6‐azophenyl‐2′4′‐disulfonic acid (U46619). If no vasorelaxation to substance P was observed, the vessel segment was excluded from further study. Then vessels were subjected to 100 mmol/L KCl to determine the maximal vascular contraction (Mulvany and Halpern 1977). Thereafter, pulmonary arteries were allowed to equilibrate in fresh Krebs solution for 30 min, before initiating different experimental protocols (Zhou et al. 2014).

### Response to vasoactive substances in vitro

After 30 min of equilibration in fresh Krebs, pulmonary small arteries were precontracted with 100 nmol/L U46619 before starting with one of five different experimental protocols (Zhou et al. 2014). Only one protocol was executed per vessel segment and within one protocol, all vessels were obtained from different animals.

In a first set of segments (Normoxia, *N* = 5 (3 female, 2 male); Hypoxia, *N* = 8 (4 female, 4 male)), endothelium‐dependent vasodilatation to bradykinin (BK; 10^−10^–10^−6.5^ mol/L; Sigma‐Aldrich, Zwijndrecht, The Netherlands) was recorded. Concentration‐response curves (CRC) to the PDE‐5 inhibitor sildenafil (10^−10^–10^−5^ mol/L; Sigma‐Aldrich) were constructed from a second set of segments (Normoxia, *N* = 6 (four female, two male); Hypoxia, *N* = 8 (four female, four male)). To study whether responses to endothelium‐independent but NO‐mediated vasodilatation were altered by transient exposure to chronic hypoxia, CRCs to exogenous NO‐donor sodium nitroprusside (SNP; 10^−9^–10^−6^ mol/L; Sigma‐Adrich) were examined (Normoxia, *N* = 9 (six female, three male); Hypoxia, *N* = 8 (four female, four male)). Finally, separate vessels segments were studied to determine whether impairments in smooth muscle cell relaxation to the NO second messenger, cyclic GMP, were involved in altered responses to SNP. For these studies, CRCs to 8‐bromo‐cyclic GMP (10^−7^–10^−3.5^ mol/L) were measured (Normoxia, *N* = 8 (five female, three male); Hypoxia, *N* = 8 (four female, four male)).

### Data analysis and statistical analysis

Digital recording and off‐line analysis of hemodynamics have been described previously (Stubenitsky et al. 1998). Pulmonary vascular conductance (PVC) was defined as CO divided by PAP minus LAP (Merkus et al. 2008). Systemic vascular conductance (SVC) was calculated as the ratio of CO and MAP. To accommodate for the varying weights between animals and groups, CO, PVC, and SVC were indexed to body weight. Statistical analysis was performed using SPSS version 21.0 (IBM, Armonk, NY). To test whether the effect of drug intervention (change vs. control; Δ) on exercise response was different in Hypoxia versus Normoxia, regression analysis was performed with FiO_2_ and treadmill speed, as well as their interaction as independent variables and animal number as case label. To test for the effect of hypoxia (vs. normoxia) on endothelium‐independent but NO‐mediated vasodilatation (SNP), General Linear Model (GLM) – Repeated Measures were used.

Vascular relaxation responses to the different vasoactive agents were expressed as percentage of contraction to U46619. Statistical analysis was performed using SPSS version 21.0 (IBM) and Prism version 5.0 (Graphpad Software, Inc., La Jolla, CA). The maximal relaxation (*E*_max_) and half maximal effective concentration (EC50) in each experiment were calculated using the GraphPad Prism version 5 for Windows (Graphpad Software, San Diego, CA). Statistical analysis of maximal relaxation and EC50 was performed using nonlinear regression (log (agonist) vs. response).

Statistical significance was accepted at *P* ≤ 0.05. Grouped data are presented as mean ± SEM.

## Results

### Effect of chronic exposure to hypoxia on hemodynamics at rest and during exercise

**Figure 2:**
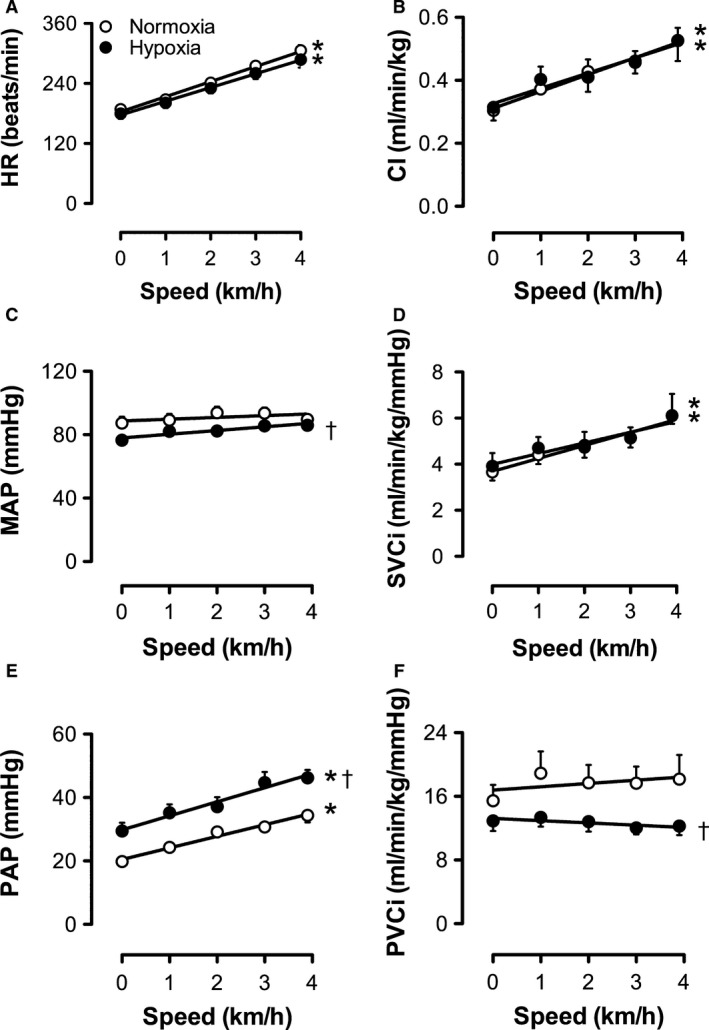
Changes in systemic and pulmonary hemodynamics during graded treadmill exercise in Normoxia (*N* = 10, 9 ± 1 day postsurgery) ‐and Hypoxia (*N* = 9, 9 ± 1 day postsurgery and re‐exposure to normoxia) exposed piglets. Relation between treadmill speed and (A) heart rate (HR), (B) cardiac index (CI), (C) mean arterial pressure (MAP), (D) systemic vascular conductance index (SVCi), (E) mean pulmonary arterial pressure (PAP) and (F) pulmonary vascular conductance index (PVCi). Values are mean ± SEM. *P ≤ 0.05 effect of exercise; †*P* ≤ 0.05 versus Normoxia.

Exercise up to 4 km h^−1^ produced a significant increase in heart rate (Fig. 2A) and cardiac index (Fig. 2B) both in animals raised in normoxia and hypoxia. Although MAP was slightly, but significantly, lower in hypoxia‐raised piglets as compared to normoxia‐raised piglets, the exercise‐induced systemic vasodilatation, as measured by the decrease in SVCi, was comparable between the two groups, and minimally affected MAP in either the normoxic or the hypoxic group (Fig. 2C and D).

In the pulmonary circulation, PVCi remained constant during exercise due to the small vasodilator capacity of the lung vasculature (Fig. 2F; normoxia, 16 ± 10%; hypoxia, −3 ± 6%). The increase in LAP during exercise, in combination with the marked increase in cardiac index, which caused an increase in the pressure drop across the pulmonary vasculature, in the face of a constant PVCi, resulted in a progressive increase in PAP with incremental levels of exercise. This exercise‐induced increase in PAP was similar in both groups (Fig. 2E).

Both at rest, and during incremental exercise, PAP was significantly higher in hypoxia‐exposed piglets as compared to normoxia‐exposed piglets (Fig. 2E). PVCi was significantly lower in animals raised in hypoxia, indicative for pulmonary vasoconstriction and/or vascular remodeling (Fig. 2F). Thus, exposure to chronic hypoxia in early life leads to pulmonary hypertension at rest and during exercise, even following re‐exposure to normoxia.

### Effect of chronic exposure to hypoxia on the NO‐pathway in the pulmonary vasculature in vivo

**Figure 3:**
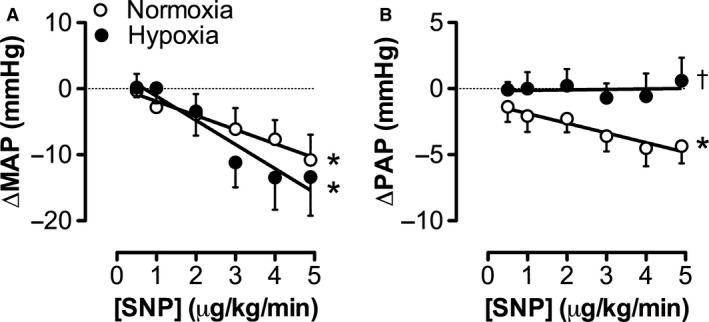
Effect of exogenous NO‐donor sodium nitroprusside (SNP) on mean arterial pressure (MAP) and mean pulmonary artery pressure (PAP) during graded treadmill exercise in normoxia‐ and hypoxia‐exposed piglets. Relation between SNP‐dosage and (A) change in mean arterial pressure (∆MAP; Normoxia, *N* = 9; Hypoxia, *N* = 8), and (B) change in mean pulmonary artery pressure (∆PAP; Normoxia, *N* = 10; Hypoxia, *N* = 8). Experiments were performed 10 ± 1 days postsurgery in both groups. Values are mean ± SEM. *P ≤ 0.05 effect of SNP‐dosage; †*P* ≤ 0.05 versus Normoxia.

In the systemic circulation in vivo, administration of incremental dosages of the exogenous NO‐donor SNP resulted in a similar decrease in mean aortic pressure in resting hypoxia‐ and normoxia‐exposed animals (Fig. 3A), while heart rate, cardiac index (CI) and SVCi did not significantly change in either group (data not shown). In contrast to the systemic hemodynamic response, SNP caused a dose‐dependent decrease in PAP only in normoxia‐exposed piglets (Fig. 3B). In hypoxia‐exposed piglets, the pulmonary vasodilator response to SNP was abolished (Fig. 3B), indicative for a reduced responsiveness of the pulmonary vascular bed to NO.

**Figure 4:**
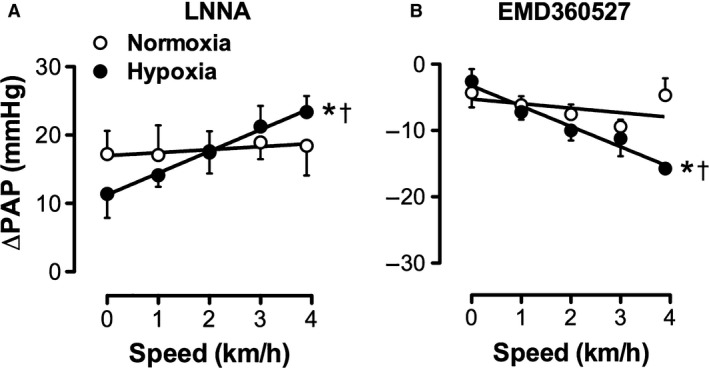
Effect of drug intervention on mean pulmonary artery pressure (PAP) during graded treadmill exercise in normoxia‐ and hypoxia‐exposed piglets. Relation between treadmill speed and (A) change in mean pulmonary arterial pressure (∆PAP) after administration of NO‐synthase inhibitor LNNA (Normoxia, *N* = 9; Hypoxia, *N* = 8), and (B) change in mean pulmonary artery pressure (∆PAP) after administration of PDE5‐inhibitor EMD360527 (normoxia, *N* = 6; Hypoxia, *N* = 5). LNNA‐experiments were performed 18 ± 1 (normoxia) and 19 ± 1 (hypoxia) days postsurgery, whereas EMD360527 experiments were performed 16 ± 2 (Normoxia) and 14 ± 1 (Hypoxia) days postsurgery. Values are mean ± SEM. *P ≤ 0.05 effect of exercise; †*P* ≤ 0.05 versus Normoxia.

Administration of the NO‐synthase inhibitor LNNA in vivo increased MAP and decreased SVCi to a similar extent in normoxia‐exposed and hypoxia‐exposed piglets, both at rest and during incremental levels of exercise (Table 2). This increase in aortic pressure was accompanied by a decrease in CI (Table 2). Similar to the systemic hemodynamic response, PAP markedly increased after LNNA administration in both groups (Fig. 4A). This increase in PAP was the result of extensive pulmonary vasoconstriction, as evidenced by a marked decrease in PVCi (Table 2). At rest, the LNNA‐induced decrease in PVCi tended to be smaller in hypoxia‐exposed piglets (Table 2, ΔPVCi normoxia vs. hypoxia p = 0.09), resulting in a trend toward a smaller increase in PAP in hypoxia‐exposed piglets as compared to normoxia‐exposed piglets (Fig. 3A; ΔPAP normoxia, +17 ± 3 mmHg; hypoxia, +12 ± 3 mmHg; p = 0.06). In contrast, the effect of LNNA on PAP during exercise increased significantly more in hypoxia‐exposed piglets as compared to controls (Fig. 4A; FiO2*exercise *P* = 0.05).

**Table 2:** Hemodynamics in normoxia‐exposed and hypoxia‐exposed piglets

	Normoxia				Hypoxia				
		Rest	Maximum exercise	Δ Rest	Δ Maximum exercise	Rest	Maximum exercise	Δ Rest	Δ Maximum exercise
HR (Beats min^−1^)	Control	171 ± 6	289 ± 13*	−24 ± 6§	−26 ± 22	174 ± 8	290 ± 13*	‐8 ± 9	−23 ± 14
	LNNA	147 ± 8‡	263 ± 19*			166 ± 10	267 ± 17*		
	Control	166 ± 21	221 ± 71	10 ± 10	−4 ± 10	140 ± 9	294 ± 18*	43 ± 18	7 ± 16
	EMD360527	176 ± 26	218 ± 69			183 ± 18	301 ± 23**		
MAP (mmHg)	Control	92 ± 4	96 ± 8	36 ± 4§	26 ± 11§§	82 ± 5	85 ± 3	30 ± 7§	36 ± 2§
	LNNA	127 ± 5‡	122 ± 15‡‡			111 ± 4^†^ ‡	120 ± 3*‡		
	Control	102 ± 11	96 ± 8	−8 ± 2§	−5 ± 7	83 ± 3	91 ± 2*	−8 ± 4^§§^	−19 ± 1*†§
	EMD360527	95 ± 11‡	91 ± 1			75 ± 5‡‡	72 ± 2^†‡^		
LAP (mmHg)	Control	4 ± 2	8 ± 3	4 ± 2§	−1 ± 3	4 ± 1	7 ± 1*	4 ± 2^§§^	2 ± 2
	LNNA	8 ± 3‡	7 ± 4			8 ± 2‡‡	9 ± 2		
	Control	4 ± 2	6 ± 4	2 ± 1	3 ± 1*§§	2 ± 1	9 ± 2*	0 ± 1	−2 ± 1**†
	EMD360527	5 ± 2	9 ± 3**‡‡			3 ± 1	7 ± 2*		
CI (L min^−1^ kg^−1^)	Control	0.39 ± 0.03	0.46 ± 0.07*	−0.11 ± 0.02§	−0.19 ± 0.04§	0.34 ± 0.06	0.51 ± 0.07*	−0.06 ± 0.03	−0.14 ± 0.03**§
	LNNA	0.28 ± 0.04‡	0.27 ± 0.06*‡			0.28 ± 0.05	0.37 ± 0.05*‡		
	Control	0.31 ± 0.04	0.44 ± 0.07*	0.00 ± 0.02	0.04 ± 0.02	0.26 ± 0.03	0.45 ± 0.04*	0.12 ± 0.12	0.15 ± 0.11
	EMD360527	0.31 ± 0.03	0.48 ± 0.09*			0.29 ± 0.02	0.60 ± 0.14**		
SVCi (ml min^−1^ kg^−1^ mmHg^−1^)	Control	4.5 ± 0.4	5.8 ± 0.5*	−2.1 ± 0.2§	−2.8 ± 0.1§	3.3 ± 0.5††	5.1 ± 0.2	−0.9 ± 1.1	−2.5 ± 0.2§
	LNNA	2.4 ± 0.3‡	2.9 ± 0.4**‡			2.4 ± 0.5	2.7 ± 0.1‡		
	Control	3.1 ± 0.1	5.3 ± 0.5**	0.3 ± 0.4	0.9 ± 0.2	3.2 ± 0.6	5.0 ± 0.5*	0.8 ± 0.1§	3.3 ± 1.5
	EMD360527	3.5 ± 0.5	6.2 ± 0.4			4.0 ± 0.6‡	8.3 ± 2.0**		
PVCi (ml min^−1^ kg^−1^ mmHg^−1^)	Control	26.5 ± 3.6	22.0 ± 7.3	−12.8 ± 2.2§	−13.1 ± 4.2§	14.6 ± 1.0^†^	14.9 ± 1.4	−2.9 ± 5.3††	−8.7 ± 1.1§
	LNNA	13.7 ± 4.5‡	8.9 ± 3.4‡			11.8 ± 4.3	6.2 ± 0.4‡		
	Control	15.9 ± 2.5	15.1 ± 3.3	10.6 ± 5.6	7.3 ± 2.3^§§^	16.6 ± 3.0	16.6 ± 4.6	4.5 ± 1.7§	10.3 ± 2.3*§
	EMD360527	26.5 ± 5.3	22.4 ± 4.1‡‡			21.1 ± 2.5‡	22.4 ± 3.5‡		

Values are means ± SEM; *N* = 9 normoxia‐exposed and *N* = 8 hypoxia‐exposed piglets in the control/LNNA group; *N* = 6 normoxia‐exposed and *N* = 5 hypoxia‐exposed piglets in the control/EMD360527 group. Please note that a flow probe was placed in only a subset of animals (Control‐LNNA *N* = 5 normoxia‐exposed and *N* = 4 hypoxia‐exposed; Control‐ EMD3605327 *N* = 5 normoxia‐exposed and *N* = 3 hypoxia‐exposed). Maximum exercise is 4 km h^−1^. HR, heart rate; MAP, mean arterial pressure; LAP, left atrium pressure; CI, cardiac index; SVCi, systemic vascular conductance indexed for bodyweight; PVCi, pulmonary vascular conductance indexed for bodyweight.

*P ≤ 0.05 effect of exercise; **P ≤ 0.10 effect of exercise; ^†^ *P* ≤ 0.05 versus normoxia; ^††^ *P* ≤ 0.10 versus normoxia; ^‡^ *P* ≤ 0.05 versus hemodynamic value during control treadmill experiment (without vasoreactive agent); ^‡‡^ *P* ≤ 0.10 versus hemodynamic value during control treadmill experiment (without vasoreactive agent); ^§^ *P* ≤ 0.05 versus no difference (vs. ∆ = 0); ^§§^ *P* ≤ 0.10 versus no difference (vs. ∆ = 0).

At rest, PDE5 inhibition with EMD360527 resulted in a decrease in MAP in normoxia‐exposed and hypoxia exposed piglets (Table 2). This decrease in MAP was accompanied by an EMD360527‐induced increase in SVCi, reaching statistical significance in hypoxia‐exposed piglets only. The effect of PDE5 inhibition during exercise on MAP was significantly larger in hypoxia‐exposed piglets as compared to normoxia‐exposed piglets, resulting in a significantly lower MAP at maximal exercise in hypoxia‐exposed piglets (Table 2). In the pulmonary circulation of normoxia‐exposed piglets, PDE5 inhibition increased PVCi and decreased PAP to a similar extent at rest and during exercise (Table 2, Fig. 4B). However, in hypoxia‐exposed piglets the EMD360527‐induced increase in PVCi was significantly augmented during graded treadmill exercise (Table 2; ∆PVCi rest vs. maximal exercise, *P* = 0.05). Consequently, while the decrease in PAP at rest tended to be smaller, the effect of PDE5 inhibition during exercise was significantly larger in hypoxia‐exposed as compared to normoxia‐exposed piglets (Fig. 4B; FiO2*exercise *P* = 0.01). These data suggest an impaired cGMP production or PDE5 activity in hypoxia‐exposed piglets at rest, which recovers during exercise.

### Effect of chronic exposure to hypoxia on the NO‐pathway in isolated pulmonary small arteries

**Figure 5:**
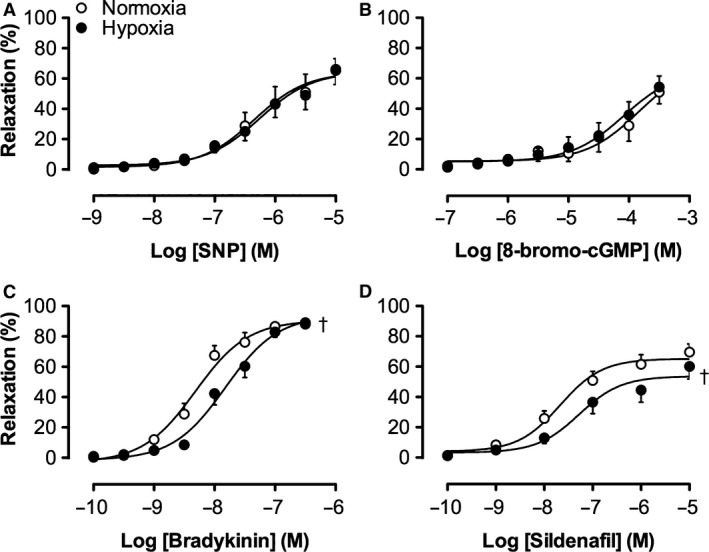
Vasodilator effects of different vasoactive agents in isolated pulmonary small arteries preconstricted with U46619 (100 nmol/L) from normoxia‐ and hypoxia‐exposed piglets. Shown are the concentration‐response curves to (A) exogenous NO‐donor sodium nitroprusside (SNP; normoxia, *N* = 9; hypoxia, *N* = 8) and (B) 8‐bromo‐cyclic GMP (Normoxia, *N* = 8; Hypoxia, *N* = 8). (C) bradykinin (Normoxia, *N* = 5; Hypoxia, *N* = 8), (D) PDE 5‐inhibitor sildenafil (Normoxia, *N* = 6; Hypoxia, *N* = 8), Values are mean ± SEM. †*P* ≤ 0.05 versus controls (vs. normoxia).

Six weeks following re‐exposure to normoxia and chronic instrumentation, responses of pulmonary small arteries were determined in vitro. There were no significant differences in concentration‐dependent vasodilatation to SNP in precontracted isolated porcine pulmonary small arteries from either normoxia‐exposed or hypoxia‐exposed piglets (Fig. 5A). Also, no differences in relaxation to 8‐bromo‐cyclic GMP, were found in pulmonary small arteries isolated from hypoxia‐exposed piglets as compared to controls (Fig. 5B).

Cumulative concentrations of bradykinin produced a concentration‐dependent vasodilatation up to 100% in precontracted isolated porcine pulmonary small arteries from both normoxia‐exposed and hypoxia‐exposed piglets (Fig. 5C). This vasodilator response to bradykinin was significantly shifted to the right in pulmonary small arteries from hypoxia‐exposed piglets as compared to normoxia‐exposed controls (logEC50 normoxia −8.32 ± 0.09 M; hypoxia −7.82 ± 0.09M; *P* < 0.05), indicative for impaired endothelium‐dependent vasodilatation in hypoxia‐exposed piglets (Fig. 5C). These findings are in agreement with the reduced vasoconstriction in the pulmonary vasculature in response to LNNA at rest in hypoxia‐exposed piglets in vivo.

In accordance with the smaller pulmonary vasodilator response in vivo in resting hypoxia‐exposed piglets, the CRC to the PDE5 inhibitor sildenafil was significantly different in precontracted isolated porcine pulmonary small arteries from hypoxia‐exposed piglets as compared to those from normoxia‐exposed piglets (Fig. 5D, logEC50 normoxia −7.67 ± 0.17 M, hypoxia −7.28 ± 0.24 M). These data are consistent with an impaired basal cGMP production or a lower PDE5 activity in pulmonary small arteries from hypoxia‐exposed piglets.

## Discussion

The present study compared the pulmonary vascular response of normoxia‐exposed control piglets and piglets with hypoxia‐induced pulmonary vascular disease, both in vivo and in vitro, to determine the functionality of different parts of the NO‐cGMP signaling pathway. The main findings of the present study were that (1) exposure to chronic hypoxia in early life leads to pulmonary hypertension at rest and during exercise, even following re‐exposure to normoxia. (2) the exogenous NO‐donor SNP produced a dose‐dependent decrease in PAP in normoxia‐exposed but not hypoxia‐exposed piglets, indicative for a reduced responsiveness of the pulmonary vascular bed to NO in this group (3) eNOS inhibition with LNNA resulted in an increase in PAP that tended to be smaller at rest, and larger during exercise in hypoxia‐exposed compared to normoxia‐exposed piglets. (4) In vivo, the PDE5 inhibition‐induced decrease in PAP tended to be smaller at rest, while the effect of PDE5 inhibition during exercise was significantly larger in the pulmonary vasculature of hypoxia‐exposed as compared to normoxia‐exposed piglets. (5) In vitro, the vasodilator response to BK was significantly shifted to the right in hypoxia‐exposed piglets and (6) the vasorelaxant response to the PDE5 inhibitor sildenafil was significantly blunted in precontracted isolated porcine pulmonary small arteries from hypoxia‐exposed piglets as compared to normoxia‐exposed piglets. However, (7) no impairments in vasorelaxation to SNP or the NO second messenger, 8‐bromo‐cyclic GMP, were found in pulmonary small arteries isolated from hypoxia‐exposed piglets as compared to controls. The implications of the se findings are discussed below.

### Animal model

Infants suffering from cardiopulmonary disorders associated with persistent or episodic hypoxia, such as BPD, are at risk for the development of PVD (including PH). Unfortunately, these patients often have a poor responsiveness to inhaled NO and alternative effective treatments for chronic PH in these patients remain largely limited. To better understand the pathophysiological mechanisms and to develop new therapies, several animal models for neonatal PVD and pulmonary hypertension, including a neonatal swine model with hypoxia‐induced pulmonary hypertension, have already been established (Plunkett et al. 1996; Scarborough et al. 1998; Perreault et al. 2001; Binns‐Loveman et al. 2005; Fike et al. 2005, 2015; Ananthakrishnan et al. 2009; Camelo et al. 2012). Swine lungs share many anatomical, histological, biochemical, and physiological features with human lungs (Rogers et al. 2008) and the relevance of the developing pulmonary circulation of neonatal piglets to human infants has been established already in the early ‘80s (Haworth and Hislop 1981, 1982). Although alveolar multiplication occurs faster in piglets (2–4 weeks compared to 3 years in human infants), the morphological development of pulmonary architecture in swine is comparable with humans (Rogers et al. 2008). We recently developed a neonatal swine model with hypoxia‐induced PH, allowing long‐term follow‐up for several weeks after re‐exposure to normoxia (de Wijs‐Meijler et al. 2017c). We showed that pulmonary hypertension induced by chronic hypoxia is transient, as pulmonary artery pressure was normalized 2–3 weeks after re‐exposure to normoxia particularly in female swine. However, despite normalization of PAP, structural and functional changes in the right ventricle and the lung vasculature (vascular remodeling with smooth muscle cell proliferation) persisted throughout the 6‐week study period (de Wijs‐Meijler et al. 2017c). In the present study, we elaborated on these findings and showed that the structural pulmonary microvascular changes were accompanied by altered regulation of pulmonary microvascular tone both in vivo and in vitro. Unfortunately, sample size in the different experimental protocols did not allow to investigate whether sex affected the vasoreactivity of the pulmonary vasculature. A limitation of our model is that the flow probe that was placed around the pulmonary artery in a subset of animals to measure cardiac output in vivo, caused a significant pulmonary artery stenosis that precluded pulmonary hemodynamic as well as right ventricular structural analyses beyond 3 weeks of re‐exposure to normoxia.

### The NO‐cGMP pathway in neonatal pulmonary vascular disease at rest

The NO‐cGMP signaling pathway is important for the adjustments in the pulmonary vasculature that accompany the transition from pre‐ to postnatal life following birth. There is increasing evidence that alterations in the NO‐cGMP signaling pathway play an important role in the pathogenesis of neonatal pulmonary vascular disease, including PH (Fike et al. 1998, 2004, 2015; Berkenbosch et al. 2000). In neonatal intensive care units, iNO is used as rescue therapy for preterm infants with respiratory disease undergoing ventilation (Berkelhamer et al. 2013b; Rossor and Greenough 2015; Baczynski et al. 2017). As perinatal hypoxia impacts the NO‐cGMP pathway (Fike et al. 1998, 2004; Herrera et al. 2010; Blum‐Johnston et al. 2016), and we have previously shown that neonatal hypoxia‐induced pulmonary vascular alterations persist for several weeks following re‐exposure to normoxia (de Wijs‐Meijler et al. 2017c), we investigated the functionality of different parts of this pathway in our model of neonatal hypoxia‐induced PH. Endothelium‐dependent vasodilatation was impaired in isolated pulmonary small arteries from hypoxia‐exposed piglets, as evidenced by a significant rightward shift of the vasodilator response to bradykinin. These data are consistent with a study showing that perinatal hypoxia results in an impaired vasodilator response to bradykinin in isolated pulmonary small arteries of lambs (Blum‐Johnston et al. 2016). We have previously shown that pulmonary vasodilation to bradykinin is largely NO‐dependent (de Wijs‐Meijler et al. 2017b), suggesting that the reduced response to bradykinin reflects impaired NO signaling. In accordance with these findings in older swine, preliminary data show that eNOS inhibition reduced the vasodilator response to bradykinin in vessels from both hypoxia‐exposed piglets (*N* = 8, logEC50 from −7.82 ± 0.09 mol/L to −7.70 ± 0.42 mol/L, maximum response from 94 ± 4% to 31 ± 7%, *P* < 0.05) and control (*N* = 2, log EC50 from −8.32 ± 0.09 mol/L to −7.29 ± 0.25 mol/L, maximum response from 91 ± 3% to 32 ± 6%, *P* < 0.05) and abrogated the difference in response to bradykinin between groups. These data are consistent with our in vivo findings that eNOS inhibition resulted in a smaller increase in PAP and PVCi at rest in hypoxia exposed piglets as well as with findings of previous studies in neonatal piglets with hypoxia‐induced PH from other groups. Both Fike et al. (1998) and Berkenbosch et al. (2000) found an impaired production of NO, through reduced eNOS protein expression and/or activity or dysfunction/uncoupling of eNOS (Fike et al. 2004, 2013, 2015) in swine following exposure to hypoxia in the early postnatal period. Timing of hypoxia and/or the animal model used may influence the effect of hypoxia on the NO‐pathway. Thus, perinatal hypoxia in lambs did not affect the contribution of NO to bradykinin‐induced dilatation (Blum‐Johnston et al. 2016).

In addition to this evidence showing an impaired NO‐production in neonatal PH, a recent study by Baczynski et al. (2017) showed that the positive response rate to iNO in preterm neonates with acute PH is only 46%, suggesting disruptions in the NO‐cGMP pathway more downstream to eNOS/NO‐production which result in an apparent reduction of the responsiveness to NO. In agreement with findings of other studies (Berkenbosch et al. 2000; Fike et al. 2013), chronic postnatal hypoxia was associated with a diminished vasodilator responsiveness to the exogenous NO‐donor SNP in vivo in the present study. In contrast, there were no significant differences in concentration‐dependent vasodilatation to SNP in precontracted isolated porcine pulmonary small arteries from either normoxia‐exposed or hypoxia‐exposed piglets. A limitation of our in vitro study is that the isolated pulmonary small arteries were used after overnight storage at 4°C, and hence it could be argued that the discrepancy between the in vivo and in vitro results may originate from this overnight storage. This is, however, unlikely as cellular processes that may affect vascular function will be decelerated at 4°C. Indeed, it has been shown that overnight storage of middle cerebral arteries does not affect vascular function to a wide variety of vasoconstrictors (prostaglandin F2*α*, UTP), endothelium‐independent (SNP, papaverine) and (partially) endothelium‐dependent, receptor‐ mediated (noradrenaline, histamine), and endothelium‐dependent, receptor‐independent eNOS‐mediated (L‐arginine) vasodilators (Laing et al. 1995). Furthermore, overnight storage is a standard procedure in our laboratory, endothelial function as assessed with substance P was preserved in the vessel segments from both normoxia and hypoxia‐exposed animals, and we have previously shown clear differences between pulmonary small arteries from swine with pulmonary hypertension secondary to pulmonary vein banding and healthy controls that were performed within a week of killing, and that were consistent with in vivo observations (van Duin et al. 2018).

An alternative explanation for the discrepancy between our in vivo and in vitro results could be that the response of one segment of the pulmonary vasculature, that is the pulmonary small arteries, in vitro does not completely reflect the response of the intact pulmonary vasculature with vessels from different sizes contributing to overall pulmonary vascular resistance responses. However, it is most likely that the discrepancy between in vivo and in vitro findings is explained by the length of re‐exposure to normoxia. In vivo experiments were performed 1–3 weeks after re‐exposure to normoxia, whereas piglets were killed after 4–6 weeks. Indeed, pulmonary small arteries from lambs exposed to prenatal hypoxia, that were maintained in normoxia for approximately 3 weeks following delivery showed a reduced responsiveness to SNP (Herrera et al. 2010). This reduced responsiveness to SNP was accompanied by upregulation of PDE5. Conversely, in our study, a reduced pulmonary vasodilator response to PDE5 inhibition in hypoxia‐exposed piglets as compared to normoxia‐exposed control piglets was present both in vivo and in vitro, which is consistent with a reduced cGMP production in piglets with hypoxia‐induced pulmonary vascular disease. The effectors of cGMP‐mediated vasodilatation are the BK_Ca_ channels. These BK_Ca_ channels are upregulated in pulmonary vascular smooth muscle cells by exposure to hypoxia in vitro (Resnik et al. 2006), and following prenatal hypoxia and postnatal normoxia in vivo (Herrera et al. 2010). However, their contribution to bradykinin‐induced vasodilatation in vitro following perinatal hypoxia was reduced (Blum‐Johnston et al. 2016). Altogether, evidence from the literature as well as the present study suggests that the impaired endothelium‐dependent vasodilatation in piglets with hypoxia‐induced PH in the first 3 weeks after re‐exposure to normoxia is due to a reduced responsiveness to NO, probably caused by altered sensitivity and/or activity of sGC, resulting is an impaired cGMP production, which may (partially) be compensated by an increased expression of BK_Ca_ channels. Our findings are consistent with previous in vivo and in vitro studies which investigated the effect of sGC activators and stimulators in acute and chronic hypoxia. Lundgren et al. (2012) showed that sGC stimulation completely reversed the pulmonary vasoconstrictor response to acute hypoxia in pigs. Weissmann et al. (2009) also found a dose‐dependent attenuation of acute pulmonary hypoxic vasoconstriction in isolated perfused mouse lung upon sGC stimulation. Furthermore, they found that administration of the sGC activator HMR1766 during exposure to chronic hypoxia reduced pulmonary hypertension, as well as right ventricular hypertrophy and structural remodeling of the lung vasculature (Weissmann et al. 2009). In addition, sGC stimulation and/or activation have been shown to inhibit or reverse the development of chronic hypoxic pulmonary hypertension in neonatal (Deruelle et al. 2006) and adult rats (Thorsen et al. 2010) and adult mice (Dumitrascu et al. 2006). Our study adds important information to these previous studies by showing that alterations in the NO‐pathway in a neonatal porcine model are still present several weeks after re‐exposure to normoxia, and we speculate that this is due to a decrease in sGC sensitivity/activity.

### The NO‐cGMP pathway in neonatal pulmonary vascular disease during exercise

Exercise resulted in an increase in pulmonary artery pressure in both normoxia and hypoxia‐exposed swine. Such exercise‐induced increase in pulmonary artery pressure is generally observed in quadrupeds, and much less in humans (Merkus et al. 2008). The main difference between the lungs of quadrupeds and humans is that the lungs of quadrupeds are located for a large part above heart level and that the entire lung is already perfused under resting conditions, whereas the large lower lung lobes of humans are at heart level, and the upper lobes are generally minimally perfused at rest. This means that recruitment of the hypo‐perfused lung lobes during exercise as occurs in humans is not possible in swine, resulting in an increase in pulmonary artery pressure with an increase in cardiac output (Merkus et al. 2008).

It is well known that the NO‐cGMP signaling pathway plays an important role in exercise‐induced pulmonary vasodilatation (Merkus et al. 2008). Given the exercise intolerance in patients with pulmonary vascular disease, it is of interest to investigate the functionality of this pathway during exercise. In the present study, we are the first to investigate the effect of exercise on NO‐cGMP signaling in a model for neonatal hypoxia‐induced pulmonary vascular disease.

In contrast to the smaller vasoconstrictor response of the pulmonary vasculature to NO‐synthase inhibition at rest, the effect of LNNA on PAP during exercise tended to be larger in hypoxia‐exposed piglets as compared to controls.

**Figure 6:**
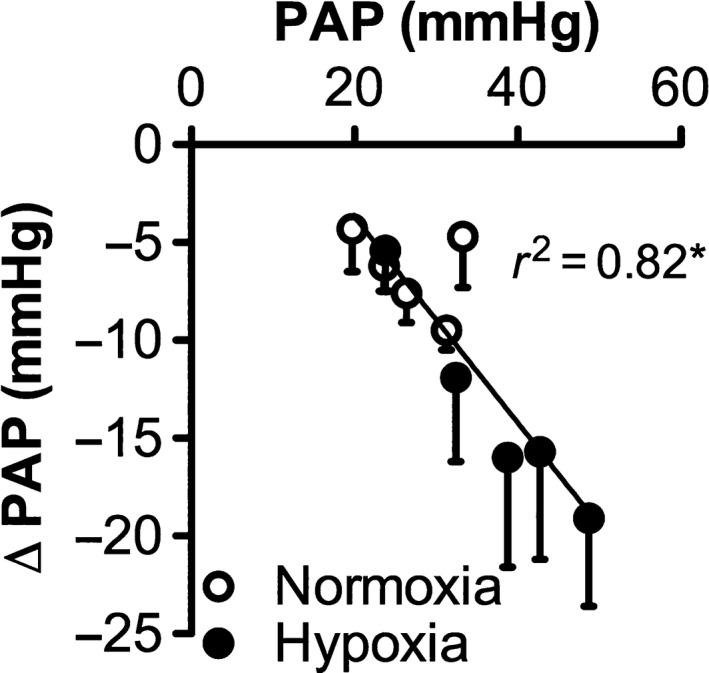
Effect of mean pulmonary arterial pressure (PA) on the pulmonary vasodilator effect of PDE5 inhibition with EMD360527. A significant correlation between PAP and the pulmonary vasodilator effect (∆PAP) of PDE5 inhibition (*r* ^2 ^= 0.82) is shown. Values are mean ± SEM. *P ≤ 0.05 correlation between PAP and ∆PAP.

Interestingly, the effect of PDE5 inhibition during exercise was significantly larger in the pulmonary vasculature of hypoxia‐exposed as compared to normoxia‐exposed piglets, whereas it tended to be smaller at rest. This apparent discrepancy between the findings at rest and during exercise suggests a normalization of cGMP production during exercise. Given the impaired sGC activity, this normalization may involve membrane‐bound or particulate guanylyl cyclase (pGC). The activity of pGC can be stimulated by natriuretic peptides (ANP and BNP) (Kobialka and Gorczyca 2000). It is possible that the significant increase in PAP during incremental exercise causes secretion of natriuretic peptides by cardiomyocytes in response to cardiac stretch (Wong et al. 2017), and thus pGC activation. In support of this hypothesis, PAP and the pulmonary vasodilator effect of PDE5 inhibition were highly correlated (*r* ^2 ^= 0.82; *P* < 0.05; Fig. 6). In hypoxia‐exposed piglets, PAP is significantly higher as compared to normoxia‐exposed piglets, resulting in higher natriuretic peptide levels and, consequently, higher pGC activity. Whether higher natriuretic peptide levels are indeed present in this animal model and act to enhance cGMP production and thereby increase the vasodilator response to PDE5 inhibition, should be tested in future experiments.

## Conclusion and Implications

In conclusion, hypoxia‐induced PH is accompanied by impaired endothelium‐dependent vasodilatation in the pulmonary vasculature. In addition to evidence for an impaired NO‐production in neonatal hypoxia‐induced PH, through a reduced eNOS protein expression and/or activity (Fike et al. 1998; Berkenbosch et al. 2000) or dysfunction/uncoupling of eNOS (Fike et al. 2004, 2013, 2015), the present study provides evidence that there are disruptions in the NO‐cGMP pathway more downstream to eNOS/NO. Thus, in our model for neonatal PH the impaired endothelium‐dependent vasodilatation was accompanied by a reduced responsiveness to NO in vivo, which may be caused by altered sensitivity and/or activity of sGC.

Our findings in a newborn animal model for neonatal pulmonary vascular disease suggests that sGC stimulators/activators could be of benefit as a novel treatment strategy to stop or even reverse neonatal pulmonary vascular disease and/or PH, especially since the use of iNO for preterm infants with respiratory failure is currently under debate (Barrington and Finer 2007; Askie et al. 2011; Cole et al. 2011; Donohue et al. 2011).

## Conflict of Interests

The authors do not have any potential or actual personal, political, or financial interest in the material, information, or techniques described in this paper.
